# Maternal Obesity, Overweight and Gestational Diabetes Affect the Offspring Neurodevelopment at 6 and 18 Months of Age – A Follow Up from the PREOBE Cohort

**DOI:** 10.1371/journal.pone.0133010

**Published:** 2015-07-24

**Authors:** Francisco J. Torres-Espinola, Staffan K Berglund, Luz Mª García-Valdés, Mª Teresa Segura, Antonio Jerez, Daniel Campos, Rosario Moreno-Torres, Ricardo Rueda, Andrés Catena, Miguel Pérez-García, Cristina Campoy, PREOBE team

**Affiliations:** 1 Centre of Excellence for Paediatric Research EURISTIKOS, University of Granada, Granada, Spain; 2 Department of Clinical Sciences, Pediatrics, Umeå University, Umeå, Sweden; 3 Department of Paediatrics, University of Granada, Granada, Spain; 4 R&D Department, Abbott Laboratories. Granada, Granada, Spain; 5 Mind, Brain and Behaviour International Research Centre (CIMCYC). University of Granada, Granada, Spain; Hôpital Robert Debré, FRANCE

## Abstract

**Background:**

Brain development in fetal life and early infancy is critical to determine lifelong performance in various neuropsychological domains. Metabolic pathologies such as overweight, obesity, and gestational diabetes in pregnant women are prevalent and increasing risk factors that may adversely affect long-term brain development in their offspring.

**Objective:**

The objective of this research was to investigate the influence of maternal metabolic pathologies on the neurodevelopment of the offspring at 6 and 18 months of life.

**Design:**

This was a prospective case-control study of 331 mother- and child pairs from Granada, Spain. The mothers were included during pregnancy into four groups according to their pre-gestational body mass index and their gestational diabetes status; overweight (n:56), obese (n:64), gestational diabetic (n:79), and healthy normal weight controls (n:132). At 6 months and 18 months we assessed the children with the Bayley III scales of neurodevelopment.

**Results:**

At 6 months (n=215), we found significant group differences in cognition composite language, and expressive language. Post hoc test revealed unexpectedly higher scores in the obese group compared to the normal weight group and a similar trend in overweight and diabetic group. The effects on language remained significant after adjusting for confounders with an adjusted odds ratio for a value above median in composite language score of 3.3 (95% CI: 1.1, 10.0; p=0.035) for children of obese mothers. At 18 month (n=197), the offspring born to obese mothers had lost five points in language composite scores and the previous differences in language and cognition was replaced by a suggestive trend of lower gross motor scores in the overweight, obese, and diabetic groups.

**Conclusions:**

Infants of obese mothers had a temporary accelerated development of cognition and language, followed by a rapid deceleration until 18 months of age, particularly of language scores. This novel observation prompts further confirmative studies to explore possible placental and neurodevelopmental mechanisms involved.

## Introduction

The rates of obesity and diabetes have experienced exceptional increase during recent years in the developed world. In parallel, studies of how these diseases affect human health are prioritized [1, 2]. One important field of research is the influence of these pathologies on pregnant women and their offspring. It is well described that excessive weight before pregnancy, particularly in combination with rapid weight gain during pregnancy, can determine the occurrence of gestational diabetes [3], a well-known risk factor both to the mother and the fetus [4]. Gestational diabetes but also pre-gestational overweight and obesity in general, have been suggested to interact with the infant micronutrient status, birth anthropometrics and even with the offspring’s neurodevelopment [4–8].

Brain development in fetus and in early years of life is critical to determine lifelong performance in various neuropsychological domains such as cognition, language, and motor functions. It has been observed that the last trimester of pregnancy is un-arguably the most important period of neuronal determination, synaptogenesis and dendritic arborization [9–11]. Thus, differences in the intrauterine environment at different stages of fetal life, may substantially determine long term neurodevelopment and brain performance [7, 9, 12, 13]. Maternal metabolic pathologies are possible examples of such differences.

In recent years, several studies have suggested that children born to mothers with gestational diabetes present language delay, impaired recognition memory, poor motor development and neuropsychological impairment at different stages of childhood [4, 8, 14–19]. Furthermore, neurodevelopment of children is associated to maternal weight gain during pregnancy, to maternal pre-pregnancy body mass index (BMI) and to obesity [5, 20–22]. However, these studies show diverging results and as recently concluded in two reviews, most of them did not prospectively explore the effects on neurodevelopment before 20 months of life [23, 24]. The effect of obesity, overweight, and diabetes on the infants’ early neurodevelopment, requires further knowledge since it may be an important predictor of psychological problems in adulthood, adult intelligence quotient (IQ), aging, and problems in executive functions [7, 25–27]. Furthermore, there is a gap in previous research considering the isolated effect of gestational diabetes and obesity respectively [23, 24].

The PREOBE study is a case control prospective cohort trial exploring pregnant women with obesity, overweight and gestational diabetes and their offspring compared to healthy, normal weight mothers. The objective of the present work was to investigate the influence of these pathologies on the offspring’s neurodevelopment at 6 and 18 months of life.

## Subjects and Methods

The general objective of this prospective cohort study was to study peri- and postnatal influence of maternal overweight, obesity and gestational diabetes. The pregnant women were recruited between 2007 and 2012 through collaboration with the two University tertiary Hospitals in Granada, the "San Cecilio" and Mother-Infant Hospital (Spain). In total, 331 healthy, pregnant women with singleton pregnancies and age between 18 and 45 years were included between 12 to 34 weeks of pregnancy. The mothers were actively included into four different groups based on their calculated pre-gestational BMI and their gestational diabetes status: Healthy normal weight group (18.5 kg/m^2^ ≤BMI<25 kg/m^2^; n = 132), overweight group (25 kg/m^2^ ≤BMI<30 kg/m^2^; n = 56), obese group (BMIx30 kg/m^2^; n = 64), and gestational diabetes group (BMI≥18.5 kg/m^2^; n = 79). The latter group was formed both by mothers included with already diagnosed gestational diabetes and by those from the first three groups who developed gestational diabetes during the study and accordingly switched group. Consequently, the gestational diabetic group included 23 with overweight and 24 with obesity. According to the present hospital routines, the mothers diagnosed of gestational diabetes were offered to participate in an endocrine-nutritional programme to optimize glucose control using nutritional and lifestyle recommendations and in some cases medical treatment. The overweight and obese mothers without gestational diabetes received no similar intervention.

**Fig 1:**
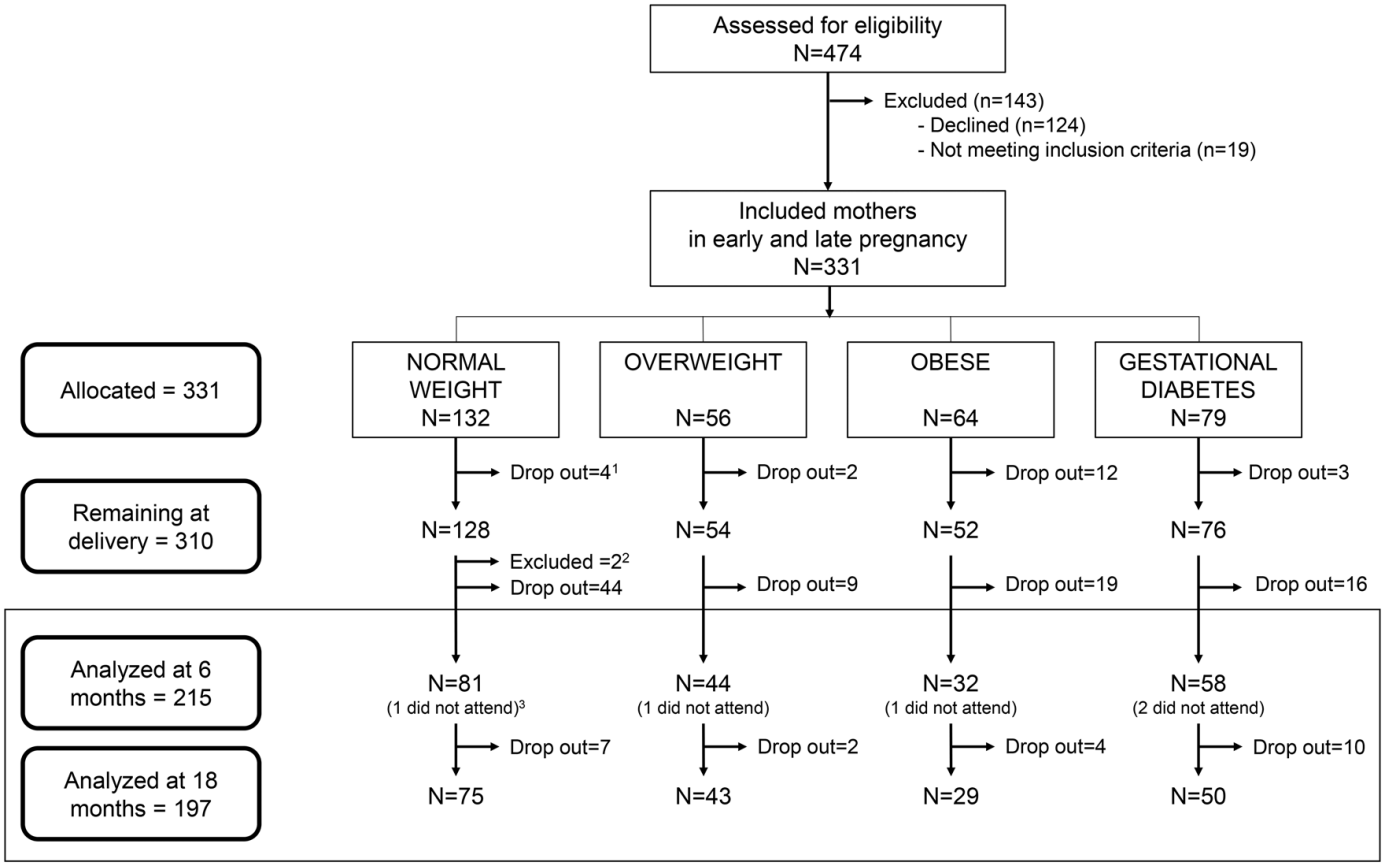
Trial profile of the neurodevelopmental follow-up. The study included 331 pregnant women at different stages of pregnancy but before 34 weeks of gestation. Included mothers were allocated to four different groups based on their pre-gestational BMI and their gestational diabetes status. ^1^In total 21 mothers dropped out of the study before delivery without giving a reason. The 310 mother-child pairs who remained at delivery constituted the four PREOBE-groups in the present trial. ^2^During early infancy, two children were excluded due to diagnosed congenital disorder (heart disease and immunodeficiency) and until 6 months, another 88 were lost to follow up giving no reason. ^3^Five children did not attend to the 6 months Bayley test but came to the test at 18 months.

The study exclusion criteria were: simultaneous participation in any other research study, any kind of drug treatment, diagnosed diseases other than obesity, overweight or gestational diabetes (e.g. pre-gestational diabetes, hypertension or preeclampsia, fetal intrauterine growth retardation, maternal infection during pregnancy, hypo/hyperthyroidism, hepatic diseases and renal disease), and vegan diet. The 310 “mother-infant” pairs who remained at delivery, defined the four final PREOBE-groups: Normal weight group (n = 128), overweight group (n = 54), obese group (n = 52) and gestational diabetes group (n = 76). In the present research, another 2 cases were excluded after delivery due to congenital disorder in the offspring (Fig 1).

Baseline and background characteristics of the pregnant women were recorded using questionnaires and medical records including age, pre-gestational weight, height and BMI, educational level, parity, smoking habits and alcohol consumption during pregnancy. Similarly, the medical records of the infants were reviewed for information regarding sex, birth weight, birth length, birth head circumference, Apgar scores, gestational age at birth, and placental weight. Furthermore at three months of age, mothers were interviewed by an expert pediatrician (AJ) about the infant diet. The pediatrician categorized the feeding mode into breastfeeding, formula or mixed type of feeding.

The present follow-up was conducted at 6 and 18 months after delivery and included an anthropometric revision, health questionnaires and medical assessments of the child. To further exclude or correct for possible confounding effects, mothers were assessed with the Intelligence test of Catell (Factor G) to obtain mothers’ IQ. Factor G is a standardized validated test available in Spanish with a population mean of 100 points and a standard deviation of 15 points.

Assessments of infant neurodevelopment at 6 and 18 months of age were conducted using the Bayley Scales of Infant Development, Third Edition (BSID-III). The BSID-III measures infant development across five domains: cognitive skills, receptive language, expressive language, fine motor, and gross motor development [28, 29]. The BSID-III was always performed in the research center by the same licensed pediatric psychologist (FJTE). The approximate total time with each test was 15–25 minutes at 6 months and 30–45 minutes at 18 months. Mothers also filled in the BSID-III questionnaire assessing the infant’s social emotional behavior.

### Ethics statement

The research was approved by the Bioethical Committees for Clinical Research of the Clinical University Hospital San Cecilio and the Mother-Infant University Hospital of Granada. An ethical approval was also obtained by the Research Bioethical Committee of the University of Granada. Written informed consent was obtained from all participants at study entry.

### Statistical analysis

All statistical analyses were performed using the SPSS statistical software package for Windows (version 20.0; IBM SPSS Inc., Chicago, IL, USA). Descriptive statistics were mean and standard deviation for normal variables, median and interquartile range for non-normal ones, and absolute frequencies and percentages for those categorical. Comparisons of continuous variables were conducted using analysis of variance (ANOVA) or in cases of non-normally distributed variables, non-parametric rank-sum test. Chi-Square test was applied to test group differences between categorical variables. In cases of significant group differences, Bonferroni corrected post hoc comparisons were used to identify significant pairwise group differences.

To assess the predictive value of the maternal pathologies, the Bayley scores were all dichotomized using the 50^th^ percentile and a logistic regression model was used to calculate the odds ratios (ORs) and 95% confidence intervals (CI) for having a value above median for the overweight group, the obese group and the gestational diabetes group respectively, using the normal weight group as a reference.

Finally, confounding effects on the Bayley outcomes were examined using the following approach: All registered baseline or background factors were compared between the groups. Variables showing significant group differences were adjusted for in univariate general linear models (ANCOVA) and in multivariate logistic regression models respectively. However, due to lack of background data in some cases, the adjusted models included fewer cases and both the unadjusted and adjusted group effects were presented.

## Results

Of the 308 mother-child pairs remaining after exclusions and drop outs at end of pregnancy, 215 were examined with the Bayley test at 6 months. At the follow up at 18 months, 5 of the missing cases came back to the study but another 23 had dropped out resulting in 197 assessed cases at the second Bayley testing (Fig 1). In Table 1, background and baseline factors were compared among the PREOBE-groups. We observed significant differences between the study groups in maternal age, weight gain during pregnancy, placental weight, maternal educational level, and as expected by group definitions, in pre-conceptional maternal BMI. The skewed variables, except BMI, were adjusted for in the analyses of group effects on the Bayley scores below. The analyzed children included 3 born late preterm, one from obese, one from overweight and one from the diabetic group. They were all assessed at corrected age.

**Table 1:** Baseline and background characteristics of the mother-child pairs who participated in the neurodevelopmental follow up at 6 months of age (n = 215), including group comparisons among the four PREOBE-groups.

		Normal weight	Overweight	Obese	Gestational Diabetes	P^1^
		*n = 81*	*n = 44*	*n = 32*	*n = 58*	
Maternal Age (y) ^*2*^		31.0±3.8^a.b^	32.7±3.9^a.c^	30.1±4.2^b^	34.0±4.2^c^	**< 0.001**
Weight Gain During Pregnancy (kg)^*3*^		13.0±5.9^a^	10.3±4.9^a.b^	7.9±6.3^b^	7.0±8.6^b.c^	**< 0.001**
Maternal educational level^4^	Primary/Secondary	42.5%^a^	55.8%^a.b^	84.4%^b^	57.9%^a^	**0.001**
	University/Doctor	57.5%^a^	44.2%^a.b^	15.6%^b^	42.1%^a^	**0.001**
Maternal IQ (points)^3^		110.1±13.4	104.2±11.4	105.4±11.4	102.9±13.2	0.074
Pre-conceptional maternal BMI (kg/m2)^*3*^		22.0±1.7^a^	27.2±1.4^b^	33.1±2.7^c^	28.0±6.6^b^	**< 0.001**
No of siblings^*4*^	0	58.8%	53.5%	40.6%	52.6%	0.387
	≥1	41.2%	46.5%	59.4%	47.4%	0.387
Smoking ^*4*^	no	83.1%	92.7%	90.3%	93.8%	0.235
	yes	16.9%	7.3%	9.7%	6.2%	0.235
Alcohol consumption during pregnancy^*4*^	No	95.8%	97.6%	96.8%	93.8%	0.827
	yes	4.2%	2.4%	3.2%	6.2%	0.827
Placental weight (g)^*3*^		483±117^a^	509±123^a.b^	560±136^b^	522.3±120^a.b^	**0.037**
Birth weight (g)^*3*^		3284±378	3290±520	3481±533	3311±408	0.176
Birth length (cm)^*3*^		50.5±1.8	50.4±1.7	51.2±2.4	50.1±2.3	0.141
Birth Head Circumference (cm)^*3*^		34.4±1.3	34.5±1.1	34.4±1.8	34.6±1.5	0.807
Sex^*4*^	Boy	49.4%^a^	45.5%^a^	53.1%^a^	55.2%^a^	0.782
	Girl	50.6%^a^	54.5%^a^	46.9%^a^	44.8%^a^	0.782
Infant type of feeding at 3 months^*4*^	Breast-fed	57.7%	53.8%	35.5%	42.3%	0.212
	Infant formula	22.5%	30.8%	48.4%	36.5%	0.212
	Mixed	19.7%	15.4%	16.1%	21.2%	0.212
Gestational Age (wk)^*3*^		39.5±1.1	39.5±1.4	39.9±1.5	39.4±1.2	0.410
Apgar 1min ^2^		9 (0)	9 (0)	9 (0)	9 (0)	0.218
Apgar 5 min^2^		10 (0)	10 (0)	10 (0)	10 (0)	0.748
Admitted to NICU^4^	No	93.5%	100.0%	100.0%	100.0%	0.053
	Yes	6.5%	0.0%	0.0%	0.0%	0.053

^1^ P-values for overall differences between PREOBE-groups. Analysis of variance for normally distributed variables, Krukal-Wallis rank-sum test for non-normal continuous variables and Chi-square test for proportions. Values who do not share the same suffix (abc) are significantly different in a Bonferroni post hoc test.

Data are:

^2^Median and Interquartile Range for non-normally distributed continuous variables:

^3^Mean ± Standard Deviation for normally distributed continues variables;

^4^Percentage for proportions.

The results of the Bayley tests at 6 and 18 months compared among the four PREOBE-groups are presented in Tables 2 and 3. At 6 months of age, we found significant group differences in the composite scores of language and cognition as well as in the subscale of expressive language. The post hoc analyses demonstrated significantly higher scores in the children born to obese mothers compared to those born to healthy normo-weight mothers. (Bonferroni corrected p = 0.013, 0.008, and 0.031 for composite language, composite cognition, and expressive language respectively). A similar, non-significant trend was observed in the overweight and diabetic group. At 18 months of age the results were different. None of the significant differences observed at 6 months remained. Instead, a significant difference was found between the study groups in the gross motor score. However, except for a *trend* of higher gross motor scores in the infants born to the normal weight group compared to the gestational diabetes group (Bonferroni-corrected p = 0.070, uncorrected p = 0.020), no pairwise group differences were significant in a Bonferroni post hoc test. The significant differences in composite language and expressive language scores at 6 months and in gross motor scores at 18 months remained in the univariate general linear model adjusting for the possible confounders (*maternal age*, *maternal education*, *placental weight*, *and weight gain during pregnancy*), but not the effect on cognition at 6 months. However, only 156 and 147 cases at 6 months and 18 months respectively were included in these models since background data were missing in some cases.

**Table 2:** Effects of maternal pre-pregnancy overweight, obesity or gestational diabetes on infant’s Bayley scores^1^ at 6 months of age compared to those born to healthy normoweight pregnant women (controls).

	Normal weight	Overweight	Obese	Gestational Diabetes	P_unadj_	P_adj_
*Bayley scores at 6 mo*	*n = 81*	*n = 44*	*n = 32*	*n = 58*		
Composite language	104.9 ± 10.1^a^	109.0 ± 9.8^ab^	111.0 ± 8.9^b^	108.1 ± 8.6^ab^	**0.009**	**0.022**
Expressive language	10.0 ± 1.9^a^	10.7 ± 2.0^ab^	11.1 ± 1.8^b^	10.4 ± 1.7^ab^	**0.024**	**0.010**
Receptive language	11.6 ± 2.2	12.2 ± 2.2	12.6 ± 2.3	12.3 ± 2.1	0.131	0.351
Composite motor	105.8 ± 11.2	104.5 ± 11.1	106.6 ± 11.1	103.7 ± 12.6	0.620	0.852
Fine motor	12.5 ± 2.2	12.4 ± 2.2	12.9 ± 1.9	12.0 ± 2.2	0.254	0.373
Gross motor	9.4 ± 2.7	9.0 ± 2.8	9.3 ± 2.4	9.2 ± 3.0	0.926	0.912
Composite cognitive	107.5 ± 7.9^a^	109.1 ± 8.0^ab^	112.8 ± 7.1^b^	108.4 ± 7.9^ab^	**0.014**	0.264
Composite socio-emotional	101.1 ± 15.6	101.2 ± 16.5	94.4 ± 18.7	99.4 ± 13.6	0.199	0.469

Data are mean ± SD

^1^ Infants’ neurodevelopment assessed using the BSID-III: Bayley Scales of Infant Development, Third Edition

P_uandj_ = Analysis of variance (ANOVA). Values not sharing the same suffix (abc) were significantly different in a Bonferroni post hoc test.

P_adj_ = Analysis of covariance (ANCOVA) for the group differences using univariate general linear model including main effects from the following possible confounder: Maternal age, maternal education, placental weight, and weight gain during pregnancy (n = 153).

**Table 3:** Effects of maternal pre-pregnancy overweight, obesity or gestational diabetes on infant’s Bayley scores^1^ at 18 months of age compared to those born to healthy normo-weight pregnant women (controls).

	Normal weight	Overweight	Obese	Gestational Diabetes	P_unadj_	P_adj_
*Bayley scores at 18 mo*	*n = 75*	*n = 43*	*n = 29*	*n = 50*		
Composite language	107.9 ± 9.3	106.2 ± 8.2	105.5 ± 8.8	106.0 ± 6.5	0.463	0.357
Expressive language	10.7 ± 1.9	10.4 ± 2.0	10.4 ± 2.1	10.4 ± 1.5	0.785	0.552
Receptive language	11.9 ± 1.8	11.9 ± 1.1	11.4 ± 1.3	11.6 ± 1.4	0.316	0.442
Composite motor	118.1 ± 10.2	115.4 ± 8.0	114.1 ± 10.0	115.0 ± 7.5	0.125	0.098
Fine motor	13.0 ± 2.3	12.9 ± 1.9	12.7 ± 2.3	12.9 ± 1.7	0.951	0.633
Gross motor	13.0 ± 2.1^a^	12.1 ± 1.9^a^	11.9 ± 1.9^a^	12.0 ± 1.9^a^	**0.015**	**0.041**
Composite cognitive	120.3 ± 11.5	123.4 ± 11.3	121.6 ± 9.6	121.8 ± 10.8	0.551	0.763
Composite socio-emotional	102.8 ± 15.2	102.8 ± 17.8	98.1 ± 17.1	102.1 ± 15.8	0.583	0.941

Data are mean ± SD

^1^ Infants’ neurodevelopment assessed using the BSID-III: Bayley Scales of Infant Development, Third Edition.

P_uandj_ = Analysis of variance (ANOVA). Values not sharing the same suffix (abc) were significantly different in a Bonferroni post hoc test.

P_adj_ = Analysis of covariance (ANCOVA) for the group differences using univariate general linear model including main effects from the following possible confounder: Maternal age, maternal education, placental weight, and weight gain during pregnancy (n = 147).

The logistic regression models, calculating the ORs for a value above median for the obese-, overweight- and gestational diabetes groups, respectively, with the normal weight group as a reference are presented in Table 4. At 6 months, the models revealed an increased chance in the obese group vs. the normal weight group of having the cognitive composite score, a composite language score, and a receptive language score above the median. A similar increased chance was seen in the overweight group with regard to composite language score. When the logistic regression models were adjusted for the four background variables, a significant group effects remained in the odds for overweight and obese group compared to the normal weight group in having a composite language score above the median.

**Table 4:** Logistic regression models assessing the odds of having the different Bayley scale scores at 6 and 18 months of age above the median. ORs are presented for infants born to mothers with overweight, obesity and gestational diabetes respectively using infants born to mothers with normal weight and no gestational diabetes as reference.

	Overweight				Obesity				Gestational Diabetes			
	*Unadjusted*		*Adjusted* ^*1*^		*Unadjusted*		*Adjusted* ^*1*^		*Unadjusted*		*Adjusted* ^*1*^	
	OR (95% CI)	P	OR (95% CI)	P	OR (95% CI)	P	OR (95% CI)	P	OR (95% CI)	P	OR (95% CI)	P
**Bayley scores at 6 months**												
Cognitive composite score > P50^2^	1.385 (0.649–2.955)	0.400	1.543 (0.628–3.793)	0.344	2.923 (1.258–6.793)	0.013*	2.310 (0.777–6.866)	0.132	1.314 (0.653–2.647)	0.444	1.407 (0.533–3.715)	0.491
Receptive language score > P50	2.038 (0.942–4.410)	0.071	1.674 (0.665–4.212)	0.274	2.366 (1.012–5.534)	0.047*	1.620 (0.536–4.900)	0.393	1.893 (0.925–3.874)	0.081	1.543 (0.571–4.169)	0.393
Expressive language score > P50	1.593 (0.761–3.336)	0.217	1.919 (0.780–4.722)	0.156	2.126 (0.924–4.891)	0.076	2.635 (0.866–8.016)	0.088	0.826 (0.412–1.654)	0.589	0.680 (0.246–1.883)	0.458
Composite language score > P50	2.375 (1.111–5.075)	0.026*	2.756 (1.099–6.910)	0.031*	2.692 (1.159–6.249)	0.021*	3.297 (1.087–10.003)	0.035*	1.676 (0.826–3.401)	0.152	1.786 (0.658–4.848)	0.255
Fine motor score > P50	1.077 (0.517–2.244)	0.843	1.233 (0.509–2.986)	0.643	1.385 (0.608–3.154)	0.438	2.029 (0.677–6.080)	0.207	0.875 (0.445–1.721)	0.699	0.762 (0.292–1.986)	0.577
Gross motor score > P50	1.262 (0.603–2.640)	0.536	1.572 (0.643–3.843)	0.321	1.075 (0.471–2.456)	0.863	1.160 (0.392–3.434)	0.789	1.382 (0.702–2.723)	0.349	1.364 (0.519–3.583)	0.529
Composite motor score > P50	0.910 (0.432–1.916)	0.804	1.058 (0.427–2.625)	0.903	1.160 (0.510–2.638)	0.724	1.137 (0.380–3.400)	0.818	0.864 (0.435–1.714)	0.675	0.701 (0.256–1.921)	0.490
Composite socio emotional score > P50	1.151 (0.543–2.441)	0.714	1.530 (0.625–3.747)	0.352	0.635 (0.261–1.546)	0.317	0.706 (0.225–2.213)	0.550	1.158 (0.563–2.382)	0.691	1.348 (0.510–3.565)	0.547
**Bayley scores at 18 months**												
Cognitive composite score > P50	2.231 (0.986–5.045)	0.054	2.457 (0.914–6.606)	0.075	1.086 (0.396–2.974)	0.873	0.978 (0.242–3.957)	0.976	1.919 (0.870–4.233)	0.106	2.337 (0.773–7.068)	0.133
Receptive language score > P50	0.648 (0.281–1.492)	0.308	0.669 (0.251–1.785)	0.422	0.393 (0.134–1.150)	0.088	0.460 (0.121–1.747)	0.254	0.532 (0.234–1.208)	0.131	0.518 (0.167–1.611)	0.256
Expressive language score > P50	0.571 (0.243–1.339)	0.198	0.626 (0.222–1.766)	0.376	0.848 (0.338–2.127)	0.725	0.678 (0.190–2.414)	0.549	0.359 (0.147–0.877)	0.025*	0.634 (0.198–2.030)	0.443
Composite language score > P50	0.904 (0.426–1.919)	0.792	1.145 (0.472–2.781)	0.764	0.480 (0.200–1.155)	0.101	0.335 (0.104–1.080)	0.067	0.442 (0.212–0.922)	0.030*	0.663 (0.244–1.796)	0.418
Fine motor score > P50	1.162 (0.537–2.516)	0.702	1.414 (0.556–3.598)	0.467	0.936 (0.381–2.300)	0.885	1.034 (0.313–3.416)	0.956	1.185 (0.567–2.475)	0.651	1.056 (0.367–3.040)	0.919
Gross motor score > P50	0.566 (0.265–1.208)	0.141	0.540 (0.217–1.344)	0.185	0.354 (0.142–0.878)	0.025*	0.333 (0.104–1.063)	0.063	0.405 (0.193–0.850)	0.017*	0.638 (0.232–1.756)	0.385
Composite motor score > P50	0.739 (0.347–1.575)	0.434	0.678 (0.271–1.693)	0.405	0.628 (0.261–1.507)	0.297	0.351 (0.107–1.157)	0.085	0.744 (0.362–1.530)	0.421	0.694 (0.251–1.919)	0.481
Composite socio emotional score > P50	1.151 (0.543–2.440)	0.714	1.002 (0.409–2.457)	0.996	0.635 (0.261–1.546)	0.317	0.974 (0.327–2.901)	0.962	1.158 (0.563–2.382)	0.691	1.641 (0.607–4.438)	0.329

All models use the *normal weight group* as reference

^1^ Logistic regression model adjusted for maternal age, maternal educational level, placental weight, and weight gain during pregnancy.

^2^ ORs for a value of Bayley score above the median.

* p< 0.05.

At 18 months, the unadjusted analyses yielded significantly lower odds for children born to mothers with pre-gestational obesity in having a gross motor score above the median and for the offspring of gestational diabetic mothers compared to normal group in having a high score of expressive language, composite language and gross motor scores. However, the differences did not remain significant in the adjusted models. Interestingly, when analyzing all groups together, a gross motor score at 18 months below or equal to median was positively associated with a composite language score above median at 6 months of age. Risk Ratio (95% CI) was 1.4 (1.1–1.7), p = 0.019.

As a last secondary analysis, the raw differences in Bayley III scores between 6 and 18 months of age were calculated for each variable and presented in Table 5. In composite language scores, the change in the obese group was significantly different compared to normal weight group (Bonferroni corrected p = 0.019). In average, the obese groups lost five points while the group of normal weight mothers showed an increase of three points.

**Table 5:** Raw difference^1^ in Bayley III scores between 6 and 18 months of age in the four PREOBE-groups.

	Normal weight	Overweight	Obese	Gestational Diabetes	P^2^
	*n = 74*	*n = 42*	*n = 28*	*n = 48*	
Composite language	3.0 ± 13.4^a^	-2.9 ± 13.0^ab^	-5.1 ± 11.7^b^	-1.5± 9.5^ab^	**0.008**
Expressive language	0.7 ± 2.6^a^	-0.4 ± 2.8^a^	-0.7 ± 2.9^a^	0.06 ± 1.9^a^	**0.043**
Receptive language	0.3 ± 2.9	-0.3 ± 2.2	-1.0 ± 2.4	-0.6 ± 2.4	0.091
Composite motor	12.0 ± 13.4	10.9 ± 13.9	7.9 ± 15.5	10.2 ± 13.7	0.601
Fine motor	0.5 ± 3.0	0.6 ± 2.4	-0.1± 3.0	0.7 ± 2.7	0.654
Gross motor	3.5 ± 2.8	2.9 ± 3.1	2.6 ± 3.2	2.6 ± 2.9	0.328
Composite cognitive	12.4 ± 12.1	14.1 ± 10.5	9.8 ± 8.8	12.7± 12.5	0.496
Composite socio-emotional	1.8 ± 14.9	1.7± 21.3	2.7 ± 18.5	0.6 ± 15.4	0.965

Data are mean ± SD.

^1^ The raw difference was calculated as the change in the score from 6 to 18 months.

^2^ P-values for overall differences between PREOBE-groups using ANOVA. Values not sharing the same suffix (abc) were significantly different in a Bonferroni post hoc test.

## Discussion

In the present case control study, we explored the effect of being born to a mother with pre-gestational overweight or obesity, or with gestational diabetes on child neurodevelopment. The unique study design made it possible to explore the effect of obesity, and overweight separated from the effect of gestational diabetes. An unexpected finding, was that children born to mothers with pre-gestational obesity had significantly higher scores in both cognitive and language development at 6 months of age. The difference was observed both when analyzed as continuous and categorical variables, even though our absolute numbers were low. Furthermore, the effect remained for language development even after adjusting for possible confounding background variables. At 18 months, the children born to obese mothers had reduced their scores significantly compared to normal weight group, and the absolute values were similar. Instead there was a minor difference between groups in gross motor scores, this time in favor of those born to mothers with normal pre-gestational weight compared to the other three groups.

Following the suggestion by Barker et al. that condition in fetal and postnatal life could contribute by *“early programming”* to later disease, several intrauterine and postnatal conditions have been identified, that associate with increased health problems and disease later in life [30]. Some of the most studied fetal conditions are maternal obesity and diabetes and how they contribute to later health in the offspring. Apart from several studies regarding risk of obesity and cardiovascular disease in the offspring, another substantial amount studies have been published about their effect on child neurodevelopment. The impact of this research field is great, considering the high and increasing prevalence of obesity and metabolic syndrome in the population. The results from previous studies are diverging and the populations examined vary. Furthermore and in contrast to the present paper, post previous research did not separate the effects of obesity, overweight and gestational diabetes respectively.

Previous published studies of the effects of maternal obesity and overweight reported impairment in various domains of neurodevelopment [5, 31–33]. Neggers et al. showed in a study of children from low income Afro-American families (n = 355, mean age 5.3 years), that obesity in the mothers predicted in a covariate adjusted model, lower child IQ and nonverbal scores compared to normal weight controls [5]. Similarly, Heikura et al. identified obesity in mothers aside with socioeconomic factors as the major risk factors for mental disability in a large Finish cohort (n>21.000) born 1986 and assessed for IQ at a mean age of 11.5 years [31], and Tanda et al. found a similar effect on cognitive scores [32]. In the largest study of the present topic, Huang et al. retrospectively analyzed over 30.000 mother-child pairs from US where the offspring were assessed by the validated intelligence test (WISC) at seven years of age. After adjusting for several background factors they showed a U-shaped association between maternal pre-pregnancy BMI and child IQ [33].

The research on the effect of obesity and overweight was reviewed in two recent papers by Van Lieshout et al 2010 and 2013. They summarized the result of in total 23 trials assessing this topic at different ages and populations. Authors concluded that the offspring of obese and overweight mothers may have an increased risk of cognitive and attention problems but the lack of data prompts further research and a causality still remains to be proven [23, 24]. A different conclusion was made in a recent paper by Brion et al. who stated that there is an association but it is confounded by sociodemographic effects [20].

The present findings at 6 months of life were unexpected and stand in complete contrast to the above reviewed literature. No previous trial has reported increased scores in any neurobehavioral assessment of obese and overweight mothers’ offspring. Considering the relatively low numbers analyzed in the present paper, and the fact that the observational design causes a risk for false positive results, we suggest caution in interpreting this finding. Nevertheless, none of the above papers assessed infants of this age and our novel findings may be, apart from a type I error, a previously not discovered early increase in neurodevelopmental abilities in this subgroup. Furthermore, a dose-response support for such effect is given by the similar, but non-significant trend in the overweight group.

The drop in neurodevelopmental scores seen in the obese group between 6 and 18 months (Table 4), particularly in language scores, suggests that the increased scores observed are, if valid, temporary and represent a short term effect. This may even indicate that the temporary increase is a part of a specific neurodevelopmental profile that in the long term is non-favorable, and a key clinical biomarker to the poorer neurodevelopmental abilities observed by others. Further support for this hypothesis is the fact that a high composite language score at 6 months positively correlated with a low gross motor score at 18 months. We can only speculate on the mechanisms behind such a temporary increase. It may be driven by increased nutrient supply from the placenta or breast milk in obese pregnancies, enabling rapid synaptogenic growth in the first months of life, but with potential negative long term effects. But our sample was small and the lack of serum samples from the growing infant limits further analyses of biomarkers for growth and neurodevelopment. The findings could be associated with differences in maternal inflammation, iron status or fatty acid profiles. However, to establish such a mechanism requires further analyses and larger sample sizes, both to confirm the hereby suggested profile of neurodevelopment, and to explore the possible mechanisms.

Our cross sectional results at 18 months are less surprising. We observed no significant differences in children born to obese and overweight mothers when analyzing continuous variables at 18 months of age, except a trend of lower scores in gross motor scores (Table 3). The logistic regressions revealed an increased risk of low motor scores in the obese group but the results did not remain significant in confounder adjusted analyses. Due to low power and the fact that most previous negative effects of overweight and obesity have been observed in later age, these results should be interpreted with caution. However, the results are opposite to 2 other previous larger trials, assessing small children with the Bayley test battery in offspring’s of maternal obesity and overweight. They both suggested significant negative effect of maternal obesity and overweight on cognitive scores but not on motor development [21, 34]. Hinkle et al. found in children born to obese mothers, a confounder adjusted reduction of 2–3 points in mental developmental index (MDI) at 2 years of age when assessed with Bayley II [34]. Similarly, Casas et al. analyzed over 2000 mother-child pairs and found a significant dose-dependent association between maternal BMI and lower cognitive scores in the offspring’s at 11–22 months of age. Obesity was associated with a decrease in Bayley cognitive scores of -2.7 and -3.7 points respectively [21]. However, none of these examined the neurodevelopment under two years of age and none explored overweight and obesity separated from gestational diabetes. Hence, our results also at 18 months therefore stand without suitable comparison to other previous research.

Effects of maternal diabetes have been targeted for repeated research during the last decades. The previous literature, suggests convincing evidence that offspring of diabetic mothers are at risk of impaired neurodevelopment [4, 15–17]. Dionne et al. compared children of diabetic mothers (n = 221) and controls (n = 2612) with regard to language development from 18 months to 7 years in two Canadian birth cohorts. They found that infants of diabetic mothers scored significantly lower in expressive language scores at several time points including at 18 months of age [17]. However, negative effects have also been reported in overall cognitive scores [15].

At 18 months follow-up, we found, in unadjusted regression analyses, that the children born to mothers with gestational diabetes had impaired scores in the domains of gross motor development but also in expressive language and composite language scores. This is in concordance with the previous literature even though the effect could not be confirmed in the subpopulation analyzed when adjusted for confounders. But we found no significant effect on cognitive scores of having a mother with gestational diabetes. This could be due to lack of power but it may also reflect the fact that the effects of diabetic mothers on development are delayed beyond 18 months of age. Actually, at 6 months the scores of cognition and language were rather higher than lower compared to the normal group, similar to the significant finding seen in the obese group. We can only speculate that the previously not observed temporary increase discussed above, also may occur in those infants born to diabetic mothers. The diabetic mothers in the present paper included obese, overweight and normal weight mothers. These subgroups may affect their offspring differently. We found no such differences (data not shown) but the study was underpowered for those analyses and the results cannot be further discussed. Further confounding effects in the gestational diabetic group is the nutritional and lifestyle recommendations that they received. These might have decreased possible adverse effects and explain why we found larger difference in the obese group.

The major limitation of our study is the large drop outs between delivery and 6 months. This caused a low study sample compared to previous research on long term effects and increased the risk of confounded results. Nevertheless, several of our observed differences remained significant in adjusted analyses and furthermore, the characteristics of our four groups were similar in most background and baseline measures, supporting that our findings, particularly on language development at 6 months, may represent a causal association. Our study was also limited by that fact that we only assess the Bayley scores. Several previous studies have reported negative effects on affective and behavioral problems, ADHD and even on anorexia nervosa, but in older children [22, 35–37]. In future follow ups of the present cohort, such outcomes need to be assessed in addition to further cognitive measures. More detailed data regarding feeding in infancy would further improve similar studies since several nutritional circumstances may cofound the results.

### Conclusions

The results obtained in this study confirmed that offspring to mothers with metabolic pathologies differ in their neurodevelopmental profile and evolution. At 18 months there was a trend of reduces scores in several domains, partly in agreement with previous research. However, we also observed a temporary increase in cognitive and language performance at 6 months, particularly in the offspring to obese mothers. This novel finding prompts further confirmative and mechanistic studies to explore the effect that the metabolic pathologies have on the fetus, the newborn and the grown up child, since it might give a clue to the pathophysiology of this increasing child health problem. We plan further follow-up of the present cohort to study the long-term effects.

## Supporting Information

S1 File Trend statement checklist. (PDF)

S2 File Study protocol. (PDF)
