# Supplementary material for: Maternal Obesity, Overweight and Gestational Diabetes Affect the Offspring Neurodevelopment at 6 and 18 Months of Age – A Follow Up from the PREOBE Cohort
Source: PLoS One. 2015 Jul 24;10(7):e0133010. doi: 10.1371/journal.pone.0133010 (PMC4514597; doi:10.1371/journal.pone.0133010)
Supplement: S2 File — (PDF) [file pone.0133010.s002.pdf]

ClinicalTrials.gov
Protocol Registration System

Protocol Registration Receipt
07/05/2012

Role of Nutrition and Maternal Genetics on the Programming of Development of Fetal Adipose Tissue (PREOBE)

This study is currently recruiting participants.
Verified by Cristina Campoy, Universidad de Granada, July 2012

Sponsor:	Universidad de Granada
Collaborators:	Spanish-Andalusian Ministry of Economy, Innovation and Science<br>Abbott Laboratories SA<br>Scottish Government NuGO EU Project<br>University of Barcelona<br>ICTAN-CSIC - Madrid - Spain<br>Ludwig-Maximilians - University of Munich<br>University of Nottingham<br>University of Aberdeen<br>General Foundation University of Granada - Enterprise<br>Agencia Andaluza del Conocimiento
Information provided by (Responsible Party):	Cristina Campoy, Universidad de Granada
ClinicalTrials.gov Identifier:	NCT01634464

Purpose

In the PREOBE projects it is aimed to obtain genetic and biochemical biomarkers for the programming of obesity in early stages of life. This can be achieved by studying pregnant women with normal weight, overweight, obesity and gestational diabetes, and by analyzing how these conditions could impact on fetal growth and development; the risk of obesity in the offspring during the first 3 years of life will be also assessed (genetic polymorphisms of the most recognized genes related to obesity; gene expression of placental biomarkers indicators of early programming, polyunsaturated fatty acids, immunological and pro-inflammatory markers, analysis of endogenous

and exogenous lipid peroxidation, allergies & neurodevelopmental assessment...). Moreover, the interaction of pathological maternal conditions with confounder factors that could have a role in the proposed outcomes will be also studied (maternal and child dietary intake, medical history and socioeconomic & environmental factors).

Condition
Obesity<br>Diabetes

Study Type: Observational
Study Design: Cohort, Prospective
Official Title: The Role of Nutrition and Maternal Genetics on the Programming of Development of Fetal Adipose Tissue. Search for Markers of the Obesity Risk in Early Stages of Life

Further study details as provided by Cristina Campoy, Universidad de Granada:

Biospecimen Retention: Samples With DNA

Maternal blood and blood from the umbilical cord, serum, plasma, placental tissue, placental tissue treated with RNAlater, saliva, oral mucosa, urine, faeces, human milk

Primary Outcome Measure:

- Long-term evidence of early human programming of obesity measured by nutritional biomarkers, and the offspring growth and development. [Time Frame: Participants will be followed for the duration of the study, an expected average of 6 years] [Designated as safety issue: No]
- Long-term evidence of early human programming of obesity measured by genetic biomarkers, and the offspring growth and development. [Time Frame: Participants will be followed for the duration of the study, an expected average of 6 years] [Designated as safety issue: No]
- Long-term evidence of early human programming of obesity measured biochemical perinatal biomarkers, and the offspring growth and development. [Time Frame: Participants will be followed for the duration of the study, an expected average of 6 years] [Designated as safety issue: No]

Estimated Enrollment: 300
Study Start Date: March 2007
Estimated Study Completion Date: June 2013
Primary Completion Date: October 2010

Groups/Cohorts	Interventions
Control group<br>women with 18.5 > BMI < 25	
Pregnant women with overweight<br>BMI≥25 before the pregnancy	
Pregnant women with obesity<br>BMI≥30 before the pregnancy	

Groups/Cohorts	Interventions
Pregnant women with gestational diabetes<br>Gestational diabetes	

Eligibility

A total of 350 pregnant women aged between 18 and 42, with singleton pregnancies, were recruited at 12 weeks of pregnancy at the Clinical University Hospital 'San Cecilio' and "Mother-Infant" Hospital in the city of Granada, Spain

Sampling Method: Non-Probability Sample
Ages Eligible for Study: 18 Years to 42 Years
Genders Eligible for Study: Female
Accepts healthy volunteers.

Inclusion Criteria:

- healthy pregnant women aged between 18 and 42
- singleton pregnancies
- pregnancy stage at recruitment: 12 weeks of pregnancy

Exclusion Criteria:

- Women who wish to participate in the study should not simultaneously participate in other research studies.
- Must be completely enclosed in one of the study of the groups without any possibility to be simultaneously incorporated on more groups of the study
- Mothers which are receiving any drug treatment, folate more than the 3rd first months, or DHA +/- vitamin supplements during pregnancy.
- Mothers affected by any disease other than those referred to the inclusion criteria, such hypertension or pre-eclampsia, foetal IUGR, mother infection during pregnancy, hypothyroidism / hyperthyroidism, hepatic diseases, renal disease,…
- Mothers following an extravagant diet or vegan diet.

Contacts and Locations

Contacts

Cristina Campoy, Professor, MD 0034 629308695 ccampoy@ugr.es

Locations

Spain

Department of Pediatrics, School of Medicine, University of Granada Recruiting
Granada, Spain, 18012
Contact: Cristina Campoy, Professor, MD 0034 629308695 ccampoy@ugr.es

Investigators

Study Director: Cristina Campoy, Professor, MD UGranada

More Information

Official website for the PREOBE Project
http://www.proyectopreobe.com

Results Publications:

Santacruz A, Collado MC, García-Valdés L, Segura MT, Martín-Lagos JA, Anjos T, Martí-Romero M, Lopez RM, Florido J, Campoy C, Sanz Y. Gut microbiota composition is associated with body weight, weight gain and biochemical parameters in pregnant women. Br J Nutr. 2010 Jul;104(1):83-92. Epub 2010 Mar 8.

Campoy C, Martín-Bautista E, García-Valdés L, Florido J, Agil A, Lorente JA, Marcos A, López-Sabater MC, Miranda-León T, Sanz Y, Molina-Font JA; grupo PREOBE. [Study of maternal nutrition and genetic on the foetal adiposity programming (The PREOBE study)]. Nutr Hosp. 2008 Nov-Dec;23(6):584-90. Spanish. PubMed

Responsible Party: Cristina Campoy, Professor, Universidad de Granada
Study ID Numbers: P06-CTS-02341
334602 Preobe Follow Up [Grant/Funding Number: Abbot Laboratories SA/General Foundation University of Granada - Enterprise]
Health Authority: Spain: Comité Ético de Investigación Clínica
